# Multi-omics of human plasma reveals molecular features of dysregulated inflammation and accelerated aging in schizophrenia

**DOI:** 10.1038/s41380-021-01339-z

**Published:** 2021-11-05

**Authors:** Anaamika Campeau, Robert H. Mills, Toer Stevens, Leigh-Ana Rossitto, Michael Meehan, Pieter Dorrestein, Rebecca Daly, Tanya T. Nguyen, David J. Gonzalez, Dilip V. Jeste, Vivian Hook

**Affiliations:** 1 Department of Pharmacology, University of California, San Diego, CA 92093 USA; 2 Skaggs School of Pharmacy and Pharmaceutical Sciences, University of California, San Diego, CA 92093 USA; 3 Amsterdam University Medical Centers, University of Amsterdam, Amsterdam, The Netherlands; 4 Sam and Rose Stein Institute for Research on Aging, University of California, San Diego, CA 92093 USA; 5 Department of Psychiatry, University of California, San Diego, CA 92093 USA; 6 Department of Neurosciences, University of California, San Diego, CA 92093 USA

**Keywords:** Schizophrenia, Prognostic markers

## Abstract

Schizophrenia is a devastating psychiatric illness that detrimentally affects a significant portion of the worldwide population. Aging of schizophrenia patients is associated with reduced longevity, but the potential biological factors associated with aging in this population have not yet been investigated in a global manner. To address this gap in knowledge, the present study assesses proteomics and metabolomics profiles in the plasma of subjects afflicted with schizophrenia compared to non-psychiatric control patients over six decades of life. Global, unbiased analyses of circulating blood plasma can provide knowledge of prominently dysregulated molecular pathways and their association with schizophrenia, as well as features of aging and gender in this disease. The resulting data compiled in this study represent a compendium of molecular changes associated with schizophrenia over the human lifetime. Supporting the clinical finding of schizophrenia’s association with more rapid aging, both schizophrenia diagnosis and age significantly influenced the plasma proteome in subjects assayed. Schizophrenia was broadly associated with prominent dysregulation of inflammatory and metabolic system components. Proteome changes demonstrated increased abundance of biomarkers for risk of physiologic comorbidities of schizophrenia, especially in younger individuals. These findings advance our understanding of the molecular etiology of schizophrenia and its associated comorbidities throughout the aging process.

## Introduction

Schizophrenia is a major psychiatric illness that affects ~1% of the population worldwide. Notwithstanding the debilitating psychiatric implications of schizophrenia, individuals suffering from this disease experience reduced lifespans of 15–20 years on average brought on by a litany of aging-related diseases, such as cardiovascular disease, diabetes, and cancer [1–4]. A pivotal development for the management of psychiatric symptoms, antipsychotic medications have a long-documented history of undesirable metabolic side effects [5]. Beyond the putative metabolic disruption caused by antipsychotics, several studies have identified a sustained pro-inflammatory molecular signature in individuals suffering from schizophrenia [6, 7]. The extra-psychotic features of schizophrenia disease presentation have led to its description as a disease of accelerated aging [8, 9].

To date, the molecular etiology and full scope of aging-related disease risk in persons with schizophrenia remains unknown. Previous studies have centered on understanding singular molecular entities (e.g., protein or metabolite) or functional pathways and their relationships to disease risk in schizophrenia. Indeed, these studies have identified inflammatory drivers associated with schizophrenia, such as Vcam1, C-reactive protein (CRP), and various cytokines [6, 10, 11]. Existing studies have also described the impact of metabolic dysregulation in schizophrenia, focusing on altered glucose and lipid metabolism stemming from both schizophrenia and antipsychotic pharmacological agents [12–14].

A growing number of investigations focus on leveraging emerging -omics technologies to uncover previously unknown mechanisms of disease and to define biomarkers associated with clinical variables [15]. For schizophrenia, such studies hold promise not only for their ability to reveal a wealth of information on the biological underpinnings of the disease, but for the new strategies they might portend for managing the metabolic and inflammatory dysfunction associated with schizophrenia and its treatment. Here, we apply contemporary untargeted mass spectrometry-based approaches to catalog proteomic and metabolomic alterations in blood plasma collected from individuals with schizophrenia and non-psychiatric subjects. The resulting data reveal a compendium of age-defined molecular factors associated with heightened disease risk in individuals with schizophrenia, including increased broad-scale inflammatory factors and metabolic dysfunction.

## Materials and methods

### Plasma sample collection

Fasting blood was collected in EDTA-treated vacutainers from 54 individuals with schizophrenia and 51 non-psychiatric comparison subjects. Subjects ranged in age from 28 to 74. There were 29 female and 25 male subjects in the schizophrenia group, and 25 female and 26 male subjects in the non-psychiatric comparison group. Following centrifugation, plasma was stored at −80 °C until assay. Standard lab assays for triglyceride, cholesterol, glucose, and insulin levels were performed by the Altman Clinical and Translational Research Institute (ACTRI) laboratory at University of California San Diego using standard procedures. The level of insulin resistance was estimated with the homeostatic model assessment of insulin resistance (HOMA-IR) = [fasting plasma insulin (mU/L) × fasting plasma glucose (mmol/L)]/22.5 [16]. High-sensitivity C-reactive protein (hs-CRP) levels were measured with a commercially available enzyme-linked immunosorbent assay (ELISA) (Meso Scale Discovery, Rockville, MD, USA). Intra- and inter-assay coefficients were < 5%.

### Protein isolation and labeling

Samples were thawed on ice and 100 μL aliquots were segregated for analysis. Samples were diluted in equal volumes of lysis buffer (3% SDS, 75 mM Sodium Chloride, 1 mM β-glycerophosphate, 1 mM sodium fluoride, 1 mM Sodium Vanadate, 10 mM Sodium Pyrophosphate, 1 mM phenylmethanesulfonyl fluoride and 1X Roche cOmplete mini EDTA free protease inhibitor in 50 mM HEPES, pH 8.5) and 8 M urea with 50 mM HEPES. Disulfide bonds were reduced in 5 mM dithiothreitol (DTT) at 56 °C for 30 min and free cysteines were alkylated in 15 mM iodoacetamide (IAA) in a darkened environment for 20 min. The alkylation reaction was quenched for 15 min at room temperature through the addition of an equivalent volume of DTT as in the reduction reaction. Protein was precipitated via the addition of 250 μL of trichloroacetic acid (TCA) and incubation of tubes on ice for 10 min. Precipitated protein was subjected to centrifugation and samples were washed twice with ice cold acetone. Protein pellets were dried and resuspended in a solution of 1 M urea with 50 mM HEPES and 50 mM ammonium bicarbonate (ABC). Protein pellets were next digested through a two-step process, wherein samples were first incubated at room temperature while shaking overnight in LysC, then incubated for 6 h at 37 °C in sequencing-grade trypsin (Promega). Supernatants were desalted on C18 columns using instructions provided by the manufacturer (Waters). Desalted samples were dried under vacuum. Samples were subjected to peptide quantification using a Pierce Colorimetric Peptide Quantification Assay kit per the manufacturer’s instructions. 50 μg of each sample was separated for further processing. An internal pooled standard sample comprised of equal masses of each sample was prepared, and 50 μg aliquots were separated for further processing.

Samples were resuspended in a solution of 30% anhydrous acetonitrile (ACN) with 200 mM HEPES, pH = 8.5. Tandem mass tag (TMT) labels (Thermo Fisher Scientific; Catalog Number: 90113) were suspended in anhydrous ACN to a final concentration of 20 mg/mL, and 7 μL were added to each resuspended sample. The labeling scheme was organized such that metadata features such as age and schizophrenia disease status were randomly and equitably represented in each TMT 10-plex. The 126 channel was reserved for the pooled internal standard across all multiplexed experiments. The labeling reaction was allowed to proceed for 1 h at room temperature, after which excess label was quenched through the addition of 8 μL of 5% hydroxylamine for 15 min. 50 μL of 1% trifluoroacetic acid (TFA) was added to each sample, and within 10-plex samples were combined and desalted on C18 columns (Waters) as above. Multiplexed samples were dried under vacuum.

Multiplexed samples were next subjected to fractionation using reverse phase high pH liquid chromatography. Briefly, samples were fractionated on an Ultimate 3000 high performance liquid chromatography system fitted with fraction collector, C18 column (4.6 x 250 mm), solvent degasser, and variable wavelength detector. Multiplexed samples were fractionated on a gradient ranging from 22% to 35% ACN with 10 mM ammonium bicarbonate (ABC) over 60 min. The resulting 96 fractions were concatenated using methods previously described [17]. Briefly, alternating wells were combined within each column, resulting in 24 total fractions. Alternating concatenated fractions were used for proteomic analysis, while the other half were used for PTM-inclusive proteomic analysis. For the “low resolution” proteome experiments, fraction A12 from ten-plexes 5 and 6 and fraction B1 for ten-plex 11 were lost during sample preparation. For the “high resolution” proteome experiments, fraction B12 from ten-plex 6 and fraction B2 from ten-plex 9 were replaced with fractions A12 and A2, respectively.

### Mass spectrometry-based proteomic analysis

All proteome mass spectrometry data were collected on an Orbitrap Fusion mass spectrometer (Thermo Fisher Scientific) with an in line Easy-nLC. Previously described methods were utilized for data collection [15]. Briefly, proteome data was collected using a “low resolution” method, wherein MS^2^ peptide fragmentation occurred in the linear ion trap. In contrast, PTM-enabled proteome data were collected using a “high resolution” method, where MS^2^ peptide fragmentation occurred in the orbitrap, reducing the overall coverage of the proteome data but enhancing our ability to match spectra to translationally modified peptides.

### Mass spectrometry-based metabolomic analysis

For metabolomic analysis, 100 μL of each plasma sample was segregated for analysis. Metabolite extraction was performed on ice, wherein 400 μL of cold methanol with 1 mM of sulfamethazine were added to each sample. Samples were subjected to mixing on a vortexer for 2 min. Protein precipitation was enhanced during an incubation period of 20 min at −20 °C. Samples were subjected to centrifugation at 16,000 × g for 15 min in order to separate precipitated protein from extracted metabolites. The supernatant resulting from centrifugation was transferred into a 96 well plate and dried under vacuum. 53 schizophrenia (SZ) and 51 non-psychiatric control (NC) samples were run on a Q-Exactive Mass Spectrometer (Thermo). Briefly, samples were run on a 150 mm (internal diameter 2.1 μm) Kinetex Polar C18 column packed with 2.6 μm particles with 100 Å pore size. Samples were run on 11.1 min gradients ranging from 0% to 100% ACN with 0.1% formic acid. Data were collected in a data-dependent fashion in positive mode. Full mass spectrometry resolution was set to 35,000 with automatic gain control target of 5 × 10^5^. The scan range was 100–500 m/z for precursor ions. For MS/MS analysis, the resolution was set to 35,000 with automatic gain control target of 5 × 10^5^. Stepped normalized collision energy levels were 20, 30, and 40. The minimum automatic gain control target was 5 × 10^3^. The apex trigger was set to 2–15 s with dynamic exclution of 10 s.

### Data processing and normalization

Raw files generated using the “low resolution” proteome method were searched using the SEQUEST algorithm in Proteome Discoverer 2.1 against the reference proteome for *Homo sapiens* downloaded from Uniprot.com on 2/18/2020. Data collected using the “high resolution” proteome method were searched using Byonic through Proteome Discoverer. “Low resolution” data were searched using a precursor mass tolerance of 50 ppm and fragment mass tolerance of 0.6 Da, while “high resolution” data were searched using a precursor mass tolerance of 20 ppm and fragment mass tolerance of 0.02 Da. For “low resolution” data, static modifications were specified as follows: TMT 6-plex on lysines and N-termini and carbamidomethylation of cysteines. Dynamic modifications were specified to include oxidation of methionines. For low resolution data, dynamic modifications were specified through Byonic using corresponding mass shifts found through Unimod.com.

For metabolomics data, the area under the curve feature abundances were calculated to produce a metabolome bucket table with the mzMine software [18]. Parameters were as follows: Mass Detection (MS^1^ noise level of 1E5, MS^2^ noise level of 1E2), ADAP Chromatogram Builder (min group size in # of scans 3, group intensity threshold 1.5E3, min highest intensity 3E5, m/z tolerance of 0.005 Da or 10 ppm), Chromatogram deconvolution (Local min search used, chromatographic threshold 0.01%, minimum in RT range 0.50 min, minimum relative height 0.01%, minimum absolute height 3E5, min ratio of peak top/edge 3, peak duration 0.05–0.50 min, m/z range for MS^2^ pairing 0.01 Da, RT range for MS2 pairing 0.10 min), Isotopic peaks grouper (m/z tolerance 0.05 m/z or 10 ppm, RT tolerance 0.10 min, maximum charge 4), Join aligner (m/z tolerance 0.005 m/z or 10 ppm, weight for m/z 90, RT tolerance 0.10 min, weight for RT 10) and filtered for at least 2 peaks in a sample and gap filling was performed to produce the final bucket table for statistical analysis. Molecular networking was performed on GNPS through feature based molecular networking (release 18) [19]. Molecular networking was performed as follows: precursor and fragment ion mass tolerance 0.05 Da, minimum cosine score of 0.7, minimum matched fragment ions of 6. Molecular class annotations were generated through the Qemistree workflow on GNPS which utilized the programs Sirius and ClassyFire [20–22]. Annotations are level 3, or family level annotations, according to the 2007 metabolomics standards intiative [23]. Data were normalized first by signal from the internal resuspension standard, sulfadimethoxine and next by the sum of the signal per sample (excluding signal from the internal standard). These percentages were then scaled to a total of 1,000,000 signal per sample for downstream analysis. Results included 1535 MS^2^ features, of which 552 had putative annotations through either GNPS or Qemistree. Data values were normalized using three steps. First, quantitation values were normalized to the quantitation value for the internal standard: sulfadimethozine. If no signal was present for this metabolite, the value was left unchanged. Next, sulfadimethoxine and its potassium derivative at 333 m/z were removed, and the values were normalized to the sum of the signal remaining in each channel. Finally, the resulting values were multiplied by 1E6.

### Data analysis with statistical assessment

Principle component analysis (PCoA) was performed using Qiime [24] for analysis of cohort proteomics data (Supplementary Tables 1 and 2). Statistical analysis testing the impact of clinical variables on mass spectrometry datasets used to construct PCoA plots were performed using PERMANOVA (for categorical variables) and Adonis (for continuous variables) [25]. Binary comparison analyses were performed in Microsoft Excel using the Student’s *T* Test with Welch’s correction when the assumption of equal variance could not be met. Altered molecules were highlighted when the binary comparison Student’s *T* Test yielded *p* < 0.05, with Benjamini–Hochberg corrected *p* values highlighted in Supplementary Table 2 (proteome). String-db analysis was performed using a compiled interaction confidence threshold of 0.6. K means clustering was performed using Morpheus (Broad Institute). Bubble plots were generated using the ‘plotly’ R package. Molecular function gene ontology analysis and Reactome analysis were performed using Gprofiler. Figures were generated using Python, Graphpad Prism, and Cytoscape. All representative images were generated using Adobe Illustrator.

As explained in this methods section, every step of the proteomics to bioinformatics procedures utilizes statistical criteria for evaluation of significance. We do, however, note that statistical significance likely, though not definitively, indicates the biological significance of the findings. Thus, the potential pitfalls of any group of methods requires that they be validated using different experimental approaches as topics of the next studies of the research program (explained at the end of the Discussion section).

## Results

### Multi-omic analysis to assess broad-scale alterations related to schizophrenia

**Fig. 1:**
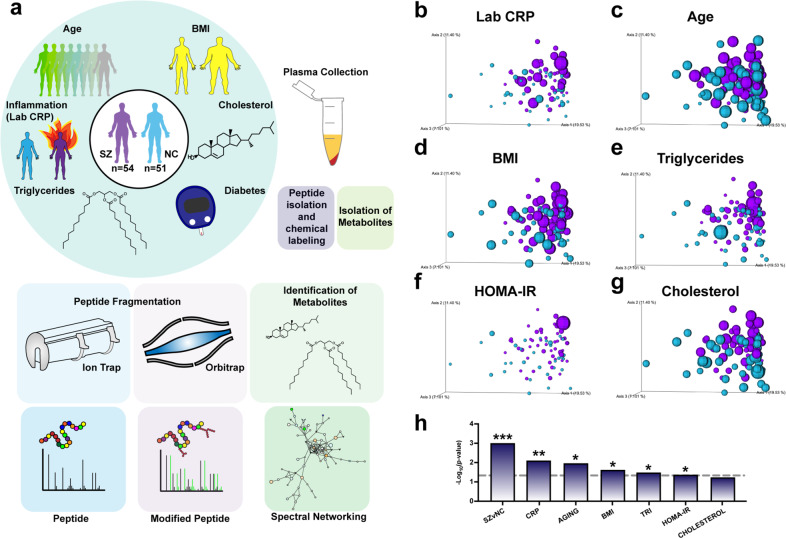
Multi-omics analyses of plasma from schizophrenia (SZ) and control non-psychiatric comparison (NC) subjects by proteomics and metabolomics to assess circulating molecular alterations. **a** Multi-omics mass spectrometry-based strategy for evaluating molecular profiles in human schizophrenia. Plasma from 54 SZ and 51 NC subjects ranging in ages 28–74 years was subjected to (i) proteomics and PTM analyses of trypsin-generated peptides subjected to TMT-labeling, fractionation, and mass spectrometry-based analysis, combined with (ii) metabolomics analyses for identification of small molecules, as described in the methods. Data on patient clinical features of inflammation (assayed by hs-CRP), BMI (body mass index), diabetes (HOMA-IR), triglycerides, and cholesterol were collected. Principal component analyses (PCoA) of metavariable influence of proteome data were conducted for (**b**) High-sensitivity C-reactive protein (hs-CRP) levels in plasma of subjects, (**c**) ages of subjects, (**d**) body mass index (BMI) of subjects, (**e**) triglyceride levels in plasma of subjects, (**f**) homeostatic model assessment for insulin resistance (HOMA-IR), and (**g**) cholesterol levels in plasma. **h** Significance measurements for metadata variable impact on PCoA distribution of proteome data. *P* values for categorical variables were measured using the PERMANOVA test for significance. *P* values for continuous variables were measured using Adonis test. Data are represented as -Log_10_(*p* value) (*p value < 0.05; **p value < 0.01; ***p value < 0.001; dotted line threshold indicates *p* value < 0.05).

In this study, we analyzed the plasma of 54 persons with schizophrenia (SZs) and 51 age-comparable non-psychiatric comparison subjects (NCs) through three mass spectrometry-based approaches analyzing proteins, post-translational modifications (PTMs) of proteins, and metabolites (Fig. 1a). Our cohort represented an equitable distribution of ages and gender within groups (Supplementary Fig. 1a, b). Sociodemographic and clinical information were also collected for these patients, demonstrating that SZs had lower levels of education, higher BMI, and higher rates of smoking (Supplementary Table 1).

Proteomic analysis quantified 742 proteins (Supplementary Table 2). PTM analysis identified 872 unique modified peptides derived from 140 proteins (Supplementary Table 3). The metabolomics data yielded 1535 metabolites, of which 159 were matched to known annotations (Supplementary Table 4).

To visualize the degree to which the collected data were influenced by clinical variables, principal component analysis (PCoA) was performed on each data set. The proteome data separated significantly by schizophrenia status (Fig. 1b–h), though the PTM-inclusive proteome and metabolome failed to do so (Supplementary Fig. 2a, b). Metadata types assessed included schizophrenia diagnosis, laboratory-measured hs-CRP level, age, body mass index (BMI), triglyceride measurement, HOMA-IR (an insulin resistance score), and cholesterol measurement. Statistical tests were used to understand the degree to which metadata variables influenced how samples differentially populated PCoA space (Fig. 1h). Interestingly, after hs-CRP measurement (Fig. 1b), the proteome data was most significantly influenced by subject age (Fig. 1c), followed by BMI (Fig. 1d), triglyceride measurement (Fig. 1e), and HOMA-IR value (Fig. 1f).

To assess the fidelity of the proteome data, CRP relative abundance values were correlated to clinical laboratory measurements, revealing a high degree of association between the datasets (SZs *p* < 0.001; NCs *p* = 0.0012) (Supplementary Fig. 1c, d). Relative abundance values of the low-density lipoprotein (LDL) marker, ApoB, were also significantly correlated to clinical LDL measurements (SZs and NCs *p* < 0.001). These findings validate the methods undertaken herein and underscore the utility of mass spectrometry-based proteomic data for elucidating the circulating plasma proteome of schizophrenia.

### Detection of metabolic dysfunction and inflammatory signatures in schizophrenia

**Fig. 2:**
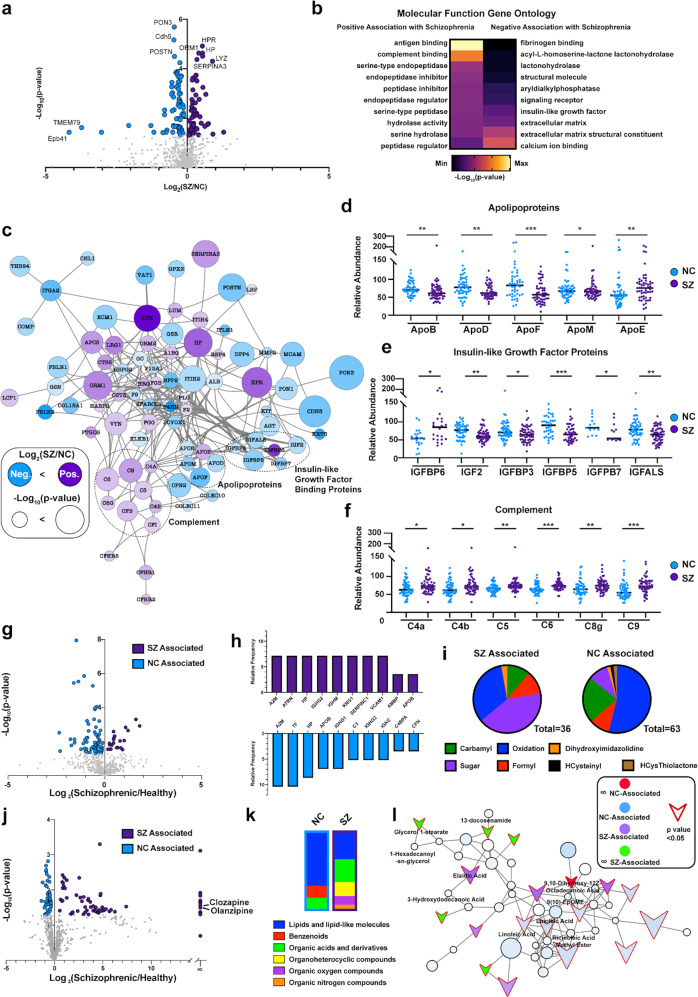
Detection of metabolic dysfunction and inflammatory signatures in schizophrenia. **a** Binary comparison of proteome data by volcano plot. The Log_2_(SZ/NC) ratios of relative abundance for proteins illustrates positive values indicating upregulation in SZ and negative values indicating upregulation in the NC controls. Proteins highlighted in purple or blue showed significant relative abundance alterations with *p* value < 0.05. **b** Molecular function gene ontology analysis of upregulated and downregulated proteins in schizophrenia compared to NC controls. Functional protein systems that are positively and negatively associated with SZ are illustrated by the color-coded heat map key. **c** String-db network of significantly altered proteins in schizophrenia patients. Network analyses conducted by String-db illustrate significantly dysregulated SZ compared to NC proteins (assessed by Log_2_(SZ/NC ratios), which include protein categories of complement, insulin-like growth factor binding proteins, and apolipoproteins. SZ and NC protein abundance profiles for (**d**) apolipoproteins, (**e**) insulin-like growth factor proteins, (**f**) complement proteins. **g** Binary comparison of PTM-enabled proteome data by volcano plot. The Log_2_(SZ/NC) ratios of relative abundance for PTM-proteins with positive values indicate upregulation and negative values indicate downregulation in SZ vs. NC controls. Proteins highlighted in purple or blue showed significant relative abundance alterations with *p* value < 0.05. **h** Relative abundance of 10 PTM-proteins with highest relative modification frequency in SZ (purple) or NC (blue) subjects. **i** Differential distribution of detected PTM types in SZ vs. NC subjects. **j** Binary comparison of metabolome data shown by volcano plot. Metabolomics data was assessed by Log_2_(SZ/NC) and -Log_10_(*p* values), illustrating SZ-associated and NC-associated metabolites. It is noted that the antipsychotic drug clozapine and olanzapine were uniquely associated with the SZ group. **k** Classification distribution of metabolites associated with NC and SZ subjects. Shown are differential distributions of proportions of identified lipid molecules, benzenoids, organic acids and related, organoheterocyclic compounds, oxygen compounds, and nitrogen compounds. **l** Dysregulated lipid molecules in schizophrenia patients assessed by GNPS spectral networks. Spectral network shown demonstrates lipid-related molecules identified by Global Natural Product Social Molecular Networking (GNPS) and their association to SZ or NC subjects. Node outline indicate significance (*p* value < 0.05), with significantly dysregulated proteins indicated by arrows.

We next sought to identify individual molecular features associated with schizophrenia. Binary comparisons were performed to identify sets of proteins increased or decreased in individuals with schizophrenia (Fig. 2a). We identified 129 upregulated and 69 downregulated proteins in our comparison of SZs to NCs. Molecular function enrichment analysis revealed a proinflammatory signature in SZs via terms such as “antigen binding” and “complement binding” (Fig. 2b).

To understand the molecular pathway relationships between the various up- and downregulated proteins, the pool of altered proteins was subjected to network analysis using the knowledge-based software tool, String-db (Fig. 2c). Proteins related to metabolism were highly interconnected, including apolipoproteins and insulin-like growth factor binding proteins (IGFBPs). Lipid-binding proteins ApoB, ApoD, ApoF, and ApoM were reduced in patients with schizophrenia, whereas ApoE was increased (Fig. 2d). Interestingly, ApoB/D/F/M have been described as constituents of LDL particles [26]. As expected, their abundance profile in schizophrenia mirrored the reduced LDL levels measured in SZs (Supplementary Fig. 1e). In contrast, ApoE, which is synthesized primarily in the brain, plays an important role in many neurological disorders including schizophrenia, where it is thought to regulate synaptic plasticity [27, 28]. Further metabolic dysregulation was detected as an upregulation in IGFBP6 in schizophrenia and a decrease in IGF2 and IGFBP3/5/7/ALS (Fig. 2e). Accompanying the metabolic disruption identified in patients with schizophrenia was an increase in several complement effector proteins, including C4a/b, C5, C6, C8a, and C9 (Fig. 2f).

Past studies have indicated a sex-bias for comorbidities in those with schizophrenia [29, 30]. Therefore, sex differences in the proteome dataset were evaluated through binary comparison (Supplementary Fig. 3a, b). Reduced angiotensinogen (AGT) in males, and increased alpha-1 acid glycoprotein (ORM1) in females were the most significant sex-specific protein features in patients with schizophrenia (Supplementary Fig. 3c, d). In the comparison of females with and without schizophrenia, 50 proteins were altered exclusively in females, 49 were altered exclusively in males, and 23 were altered in both sexes (Supplementary Fig. 3f). To visualize interaction-based relationships between the subset of proteins altered in schizophrenia in either sex, data were subjected to functional network analysis (Supplementary Fig. 3e). Interestingly, proteins altered in both sexes were highly correlated, indicating a core set of molecular changes governing adverse health outcomes of schizophrenia regardless of sex (Supplementary Fig. 3g).

Given the changes detected in proteins related to metabolic function in schizophrenia and previous reports underscoring the role of plasma protein PTMs on health and aging, we sought to determine whether schizophrenia was associated with alterations in PTMs [5, 31, 32]. Roughly two-thirds of the altered modified peptides were higher in NCs than in SZs (Fig. 2g, h). Among the modifications identified, oxidation was highly represented in differentially modified peptides in NCs. This was surprising, as protein oxidation is thought to be a prognosticator of aging and chronic illnesses of dysfunctional metabolism [33–35]. In contrast, increased peptide N-glycosylation was more highly represented in SZs than in NCs (Fig. 2i). This finding aligns with previous reports that glycosylated proteins are elevated in cardiovascular disease, a significant cause of early mortality in people with schizophrenia [1, 2, 32, 36, 37].

We next searched for altered metabolites in schizophrenia (Fig. 2j). NC- and SZ-associated metabolites were broadly classified into functional groups (Fig. 2k). The majority of altered metabolites were classified as lipids, suggesting that schizophrenia is associated with dysfunctional circulating lipid profiles. To explore functional relationships between identified metabolites, molecular networking was performed (Supplementary Fig. 4) [19, 38]. Fold change and significance data were overlaid onto the networked metabolites, revealing a network of lipid-related molecules with strong associations to schizophrenia (Fig. 2l). The annotations are level 3, or family level annotations, according to the 2007 metabolomics standards intiative [23]. Notably, the annotation from a spectral match to elaidic acid, reveals that this match to a singly unsaturated 18:1 fatty acid with a molecular formula of C_18_H_24_O_2_ (Level 3), was upregulated in SZs. In contrast, the annotation with a spectral match to linoleic acid, which represents an 18:2 fatty acid with two double bonds (C_18_H_32_O_2_, Level 3), was upregulated in NCs [39]. 18:1-fatty acids like elaidic acid have been linked to increased risk of cardiovascular disease and cancer, while 18:2-fatty acids such as linoleic acid are associated with protection from cardiovascular disease and have been linked to the pathophysiology of schizophrenia [39–44]. Taken together, these findings detail a circulating metabolic and inflammatory molecular signature associated with schizophrenia.

### Identifying molecular determinants of age-related disease risk using a machine learning strategy

**Fig. 3:**
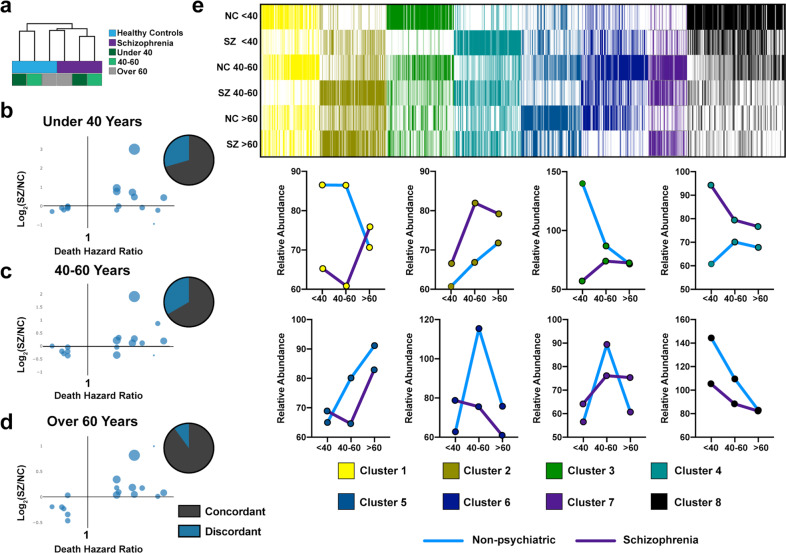
Identifying molecular determinants of age-related disease risk using a machine learning strategy. **a** Hierarchical clustering of proteome data for age-related categories in schizophrenia and healthy control subjects revealing clustering by schizophrenia status and age. Purple: schizophrenia; blue: healthy controls; dark green: < 40; mint green: 40–60; gray: > 60. Proteins associated death in ages groups of (**b**) < 40 years, (**c**) 40–60 years, and (**d**) > 60 years old. Log_2_(SZ/NC) ratios of proteins associated with death at different age categories are illustrated. **e** Age-stratification of differentially expressed protein clusters in SZ and control NC subjects. The heatmap color key shows white for relative minimum value per protein, and relative maxima are indicated by cluster-specific colors for clusters 1–8.

Given the documented risk of early death in schizophrenia and the significant impact of aging on our proteome data, we evaluated age-specific patterns of protein abundance (Fig. 1c, h). Patients were stratified into three groups representing youth, middle age, and advanced age—under 40, 40–60, and over 60—respectively, for both SZs and NCs. To validate this classification system, hierarchical clustering was performed on average protein abundances (Fig. 3a). Using this method, SZs clustered together. Interestingly, NCs over 60 also clustered with SZs, while younger NCs clustered apart. This suggests commonality between the plasma proteome of SZs at all ages with NCs in the advanced age group.

Because patients with schizophrenia experience a higher likelihood of early death, we sought to evaluate the age-stratified data against a published dataset of proteins associated with risk of death [45]. There was high concordance overall between protein abundance fold change in schizophrenia and previously reported risk of death among all age strata (Fig. 3b–d).

To uncover age-related patterns in our data, K means clustering was performed on average protein abundance from each of the six groups (Supplementary Table 5, Fig. 3e). Of particular interest was cluster 4, wherein average abundance values increased with age in healthy patients, but were higher in individuals with schizophrenia throughout the life stages, especially in individuals under 40. We hypothesize that this cluster represents a subset of proteins associated with early onset of age-related disease in SZs, as these proteins were found increased among older NCs.

**Fig. 4:**
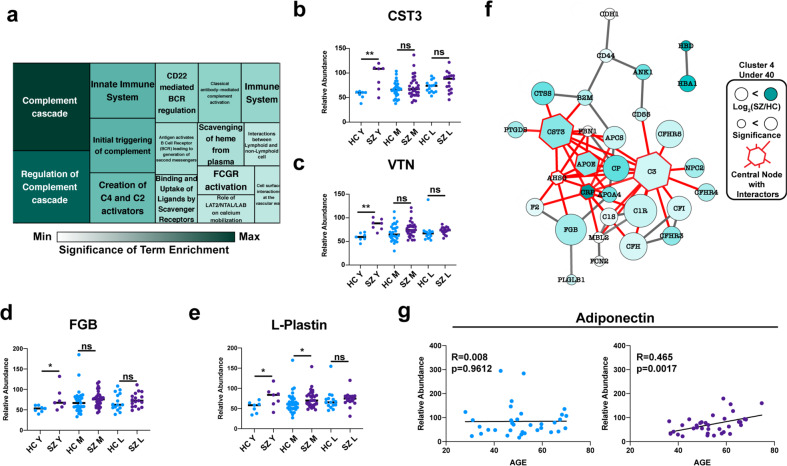
Targetable determinants of early morbidity in schizophrenia. **a** Reactome functional analysis of proteins from cluster 4 represented as a treemap. **b**–**e** Representative disease risk biomarkers identified in cluster 4. **f** String interaction network of proteins from cluster 4 (top 5 most interconnected proteins are highlighted in red with all interaction partners). **g** Trend in adiponectin protein abundance at various ages.

The proteins within this cluster were subjected to functional analysis (Fig. 4a). “Complement cascade” was the most significantly enriched term. Interestingly, there have been several reports on the relationship between complement signaling and cardiovascular disease [46–50]. Also in cluster 4 were Cystatin-3 (CST3) (Fig. 4b) and Vitronectin (VTN) (Fig. 4c), proteolysis inhibitors which have been described as robust biomarkers of heart disease [45, 51]. We identified Fibrinogen-B (FGB) (Fig. 4d), a protein involved in the clotting cascade, and L-Plastin (Fig. 4e), a critical inflammatory marker [52].

Given the heightened risk of early death in patients with schizophrenia, strategies for early intervention against dysregulated protein targets are needed. To evaluate the targetability of proteins with increased abundance associated with schizophrenia under 40, proteins from cluster 4 were subjected to network analysis through String-db (Fig. 4f). The five most highly connected proteins in this network were C3, CST3, AHSG, CRP, and ApoE. Interestingly, investigational and FDA-approved drugs have been developed to target these proteins [53–55]. These drugs represent a promising avenue for early interventions against the comorbidities associated with schizophrenia.

Finally, we hypothesized that molecular alterations in older SZs could be signs of a robustly protective plasma milieu, simply by virtue of this group’s survival into older adulthood. Indeed, adiponectin, an adipokine that regulates insulin sensitivity and may play a role in improving cognition, displayed a positive relationship with age in the SZ group (Fig. 4g) [56].

## Discussion

Schizophrenia is a severe mental illness associated with significant disruption to daily life, but also associated with physiologic comorbidities thought to contribute to a shortened lifespan. To achieve a global perspective on the circulating molecular factors contributing to altered physiology in individuals with schizophrenia, we undertook a mass spectrometry-based multi-omic analysis of blood plasma samples collected from individuals diagnosed with schizophrenia (SZs) and non-psychiatric control subjects (NCs) representing individuals from six decades of life. Our study found that despite significant heterogeneity in clinical symptoms of schizophrenia, multi-omic analysis revealed sustained metabolic disruption and increased inflammatory markers among detected circulating proteome components. For example, we identified reduced levels of ApoB/D/F/M in the proteome data, which align with clinical findings of reduced lipid measurements in schizophrenia (Supplementary Fig. 1e, f). In contrast, ApoE, a protein that was increased among SZs in our data, has been linked to the etiology of schizophrenia, but its role in metabolic health highlights this protein as a target for further investigation [27, 28]. In addition to metabolic dysfunction, we also noted increased immune-related proteins in SZs compared to NCs, a finding often correlated with increased risk of age-related diseases such as cancer, cardiovascular disease, and diabetes.

Our analysis of PTMs revealed reduced levels of oxidized peptides in schizophrenia. Oxidation often occurs as a result of oxidative stress, a process thought to increase the risk of aging-related diseases. Interestingly, in spite of decreased protein oxidation in schizophrenia, patients showed higher levels of inflammatory markers and higher overall BMI, indicators of poorer overall health. In contrast, increased N-glycosylation was higher in SZs. This finding matches previous reports highlighting glycosylation as a risk factor for atherosclerosis and cardiovascular disease [37].

Age-based stratification method revealed high levels of several known cardiovascular disease biomarkers in SZs under 40. This suggests that the molecular features of increased risk of age-related comorbidities and death in people with schizophrenia are present early in life. Highly interconnected proteins identified within this network could represent attractive targets for the prevention of comorbidities in younger patients with schizophrenia. For example, the most highly interconnected protein in the constructed network, C3, has been targeted using the long-acting C3 inhibitor, polyethylene glycol-Cp40, in paroxysmal nocturnal hemoglobinuria [53].

While the psychiatric symptoms of schizophrenia can be managed by current antipsychotic medications, there is room for improvement in the management of other aspects of the disorder, such as the cognitive deficits and significantly reduced lifespan seen in persons with schizophrenia. Despite the presence of the antipsychotic drug medications in the serum of the schizophrenia samples evaluated, there are still prominent changes in serum biomarker proteins in schizophrenia, especially those of the immune system. The data clearly shows that antipsychotic drugs are not capable of attenuating numerous dysregulated systems in schizophrenia, including, for example, cystatin C which is upregulated in SZs under 40 and is correlated with cognitive deficits [57]. It is known that antipsychotics fail to ameliorate cognitive dysfunction in schizophrenia [58, 59]. Therefore, this study reinforces the inflammatory changes in schizophrenia that are not ameliorated by antipsychotic drugs. These data demonstrate that future new approaches to address cognitive deficits, metabolic dysfunction, and increased inflammatory signaling in schizophrenia are clearly needed.

The analyses presented here lay the groundwork for better understanding metabolic disease risk factors at various stages of life in schizophrenia. Future studies should include prospective longitudinal investigations of larger and more diverse samples of SZs and NCs. Larger sample sizes will enable analyses on subgroups of patients with different illness severity and comorbidity and the development of more targeted strategies for managing schizophrenia.

## Supplementary information

Supplemental Figures 1-4

Supplemental Table 1 Cohort

Dataset 1: Supplemental Table 2 Proteomics

Dataset 2: Supplemental Table 3 PTM

Dataset 3: Supplemental Table 4 Metabolomics

Dataset 4: Supplemental Table 5 Clustering

## Data Availability

Proteome data was uploaded to massive.ucsd.edu and can be accessed through ProteomeXchange using the identifier PXD024474. The metabolomics data can be accessed through massive.ucsd.edu using the following identifier: MSV000086975.
