# Supplementary material for: Multi-omics of human plasma reveals molecular features of dysregulated inflammation and accelerated aging in schizophrenia
Source: Mol Psychiatry. 2021 Nov 5;27(2):1217–25. doi: 10.1038/s41380-021-01339-z (PMC9054664; doi:10.1038/s41380-021-01339-z)
Supplement: Supplementary file 1 — Supplemental Figures 1-4 [file 41380_2021_1339_MOESM1_ESM.pdf]

Supplemental Figures 1-4 for:

Multi-omics of Human Plasma Reveals Molecular Features of Dysregulated Inflammation and Accelerated Aging in Schizophrenia

Anaamika Campeau[1,2], Robert H. Mills[1,2], Toer Stevens[3], Leigh-Ana Rossitto[1,2], Michael Meehan[2], Pieter Dorrestein[1,2], Rebecca Daly[4,5,6], Tanya T. Nguyen[4,5,6], David J. Gonzalez[1,2*#], Dilip V. Jeste[4,5,6*], Vivian Hook[1,2,6*]

[1]Department of Pharmacology, University of California, San Diego, California 92093, USA
[2]Skaggs School of Pharmacy and Pharmaceutical Sciences, University of California, San Diego, California 92093, USA
[3] Amsterdam University Medical Centers, University of Amsterdam, Amsterdam, The Netherlands
[4]Sam and Rose Stein Institute for Research on Aging, University of California San Diego, California 92093, USA
[5]Department of Psychiatry, University of California, San Diego, California 92093, USA
[6]Department of Neurosciences, University of California San Diego, California 92093, USA
[*]Co-senior authors
[#]Corresponding author: David J. Gonzalez (djgonzalez@ucsd.edu

Supplemental Figure 1: Representative clinical variables for SZ subjects and NC subjects

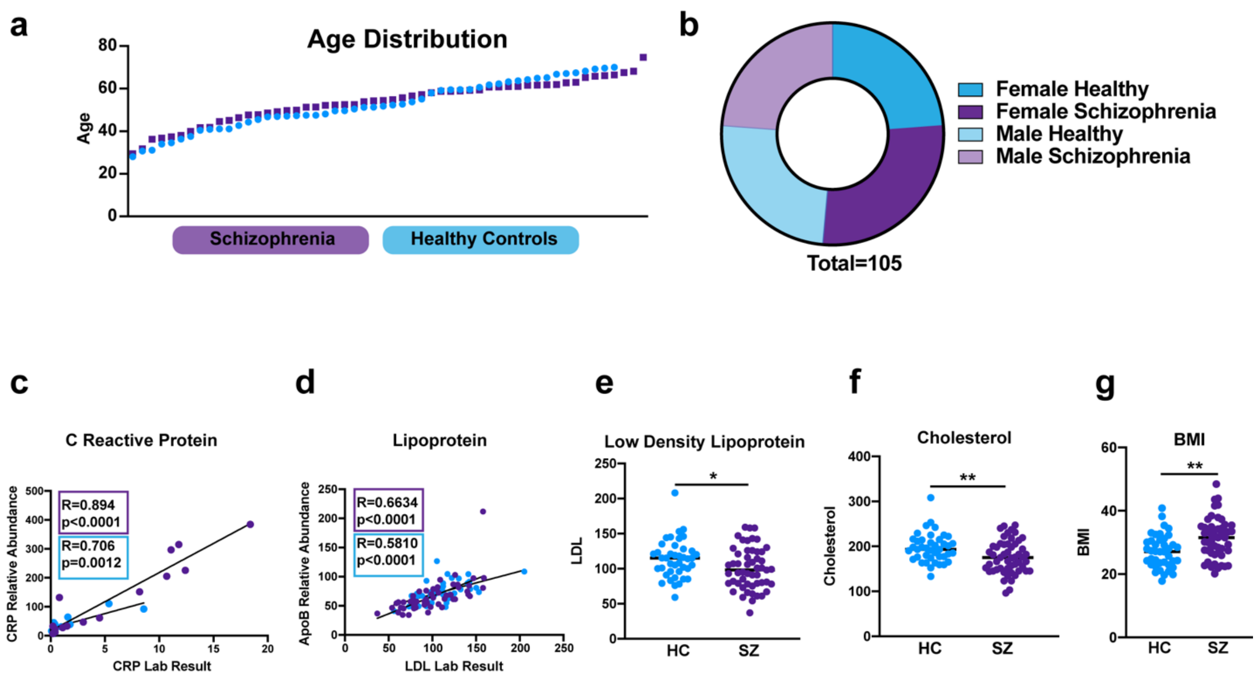

a. Age distribution of schizophrenia and healthy control subjects included in study.
b. Sex distribution of schizophrenia and healthy control subjects included in study.
c. Correlation between hs-CRP measured in the clinical laboratory and CRP relative protein abundance for schizophrenia and healthy control subjects.
d. Correlation between LDL measured in the clinical laboratory and ApoB relative protein abundance for schizophrenia and healthy control subjects.
e. LDL levels for schizophrenia and healthy control subjects included in study.
f. Cholesterol levels for schizophrenia and healthy control subjects included in study.
g. BMI measurements for schizophrenia and healthy control subjects included in study.

Supplemental Figure 2. PCoA analysis of schizophrenia's effect on PTM-enabled proteome data and metabolome data

a **b**

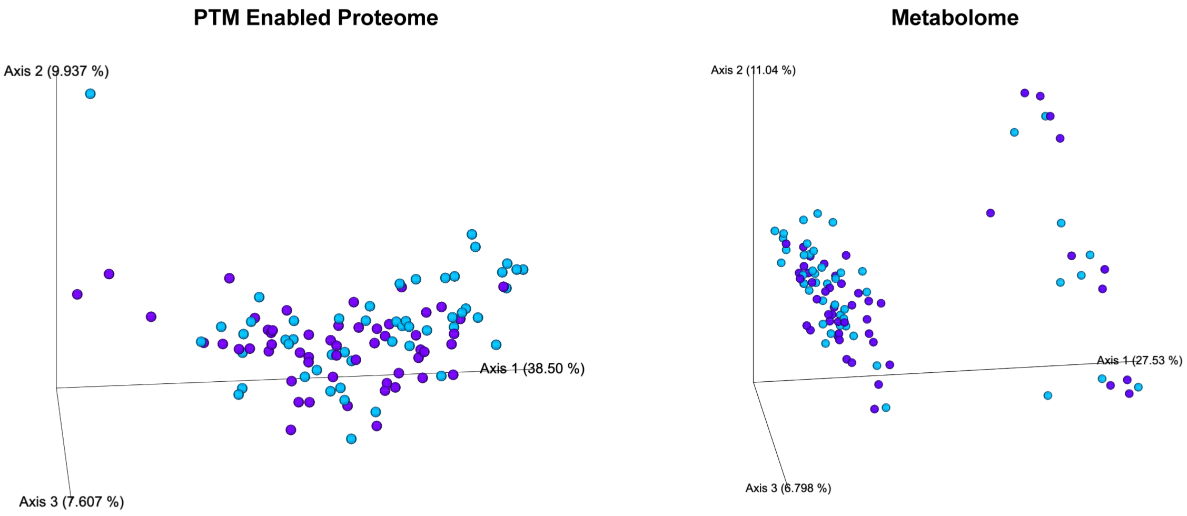

a. PCoA plot of PTM enabled proteome data, with subject data points colored by schizophrenia status (purple: SZ; blue: NC).
b. PCoA plot of metabolome data, with subject data points colored by schizophrenia status (purple: SZ; blue: NC).

Supplemental Figure 3: Sex-specific circulating proteome changes in schizophrenia

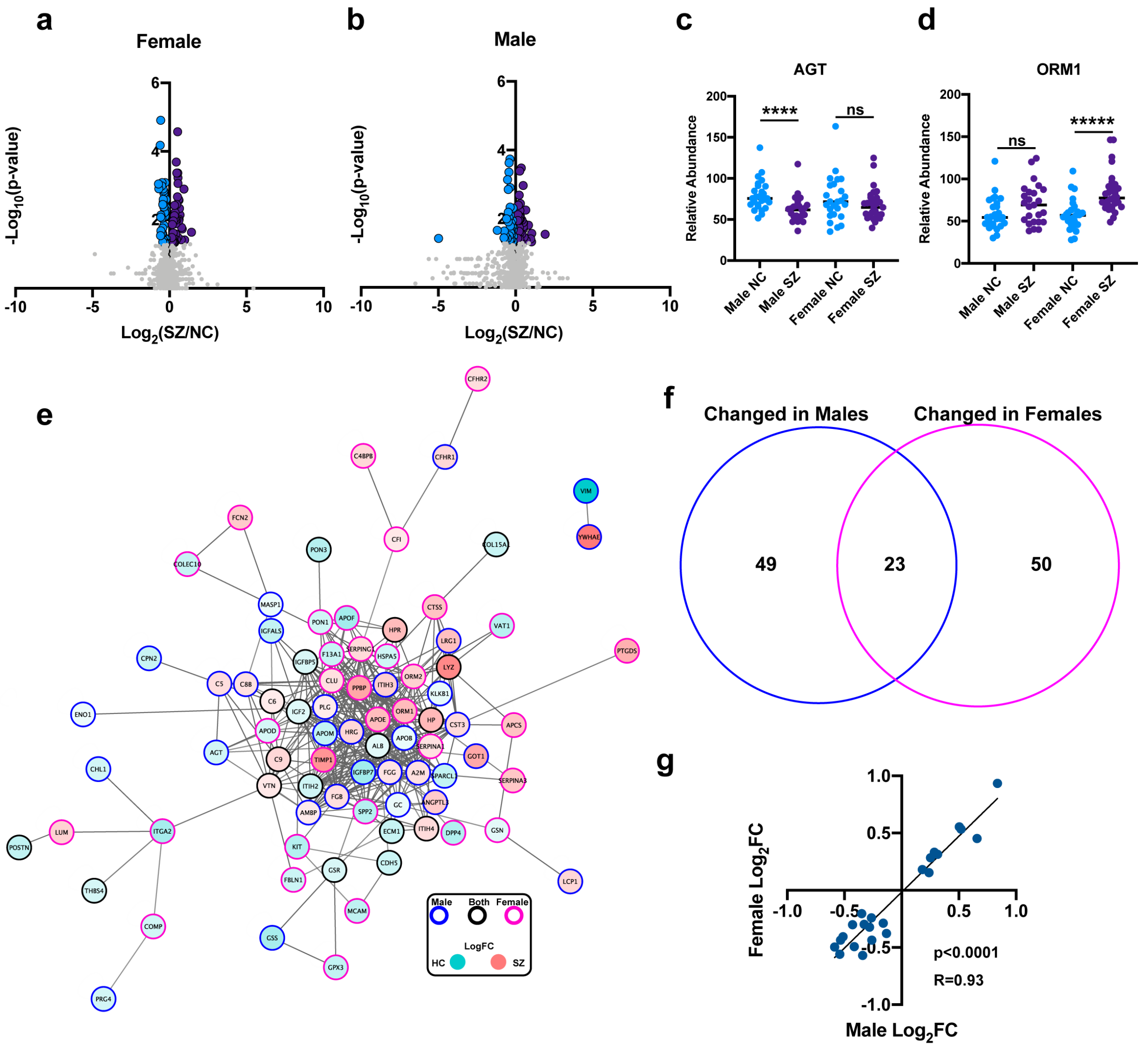

a. Volcano plot of significantly altered proteins in schizophrenia compared to healthy controls among females.
b. Volcano plot of significantly altered proteins in schizophrenia compared to healthy controls among males.
c. AGT, the most significantly altered protein in males that was not significantly altered in females.
d. ORM1, the most significantly altered protein in females that was not significantly altered in males.
e. String-db network showing interaction-based relationships between significantly altered proteins in males and females.
f. Venn diagram showing overlap of protein changes associated with schizophrenia in males and females.
g. Correlation of significantly altered proteins identified in both males and females.

Supplemental Figure 4: Detection of schizophrenia-associated metabolites through spectral networking

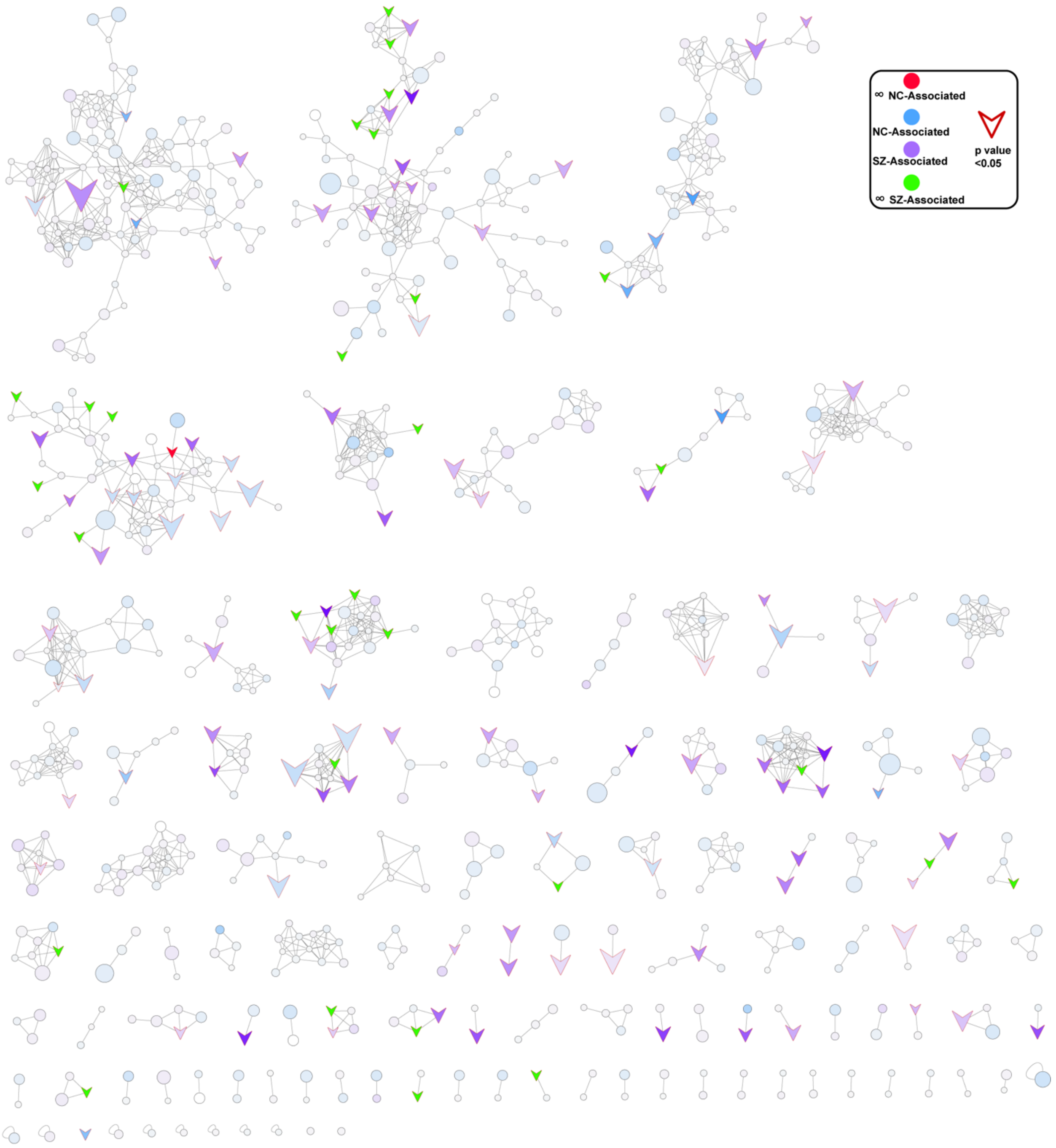

Spectral networking of identified metabolites. Colors are scaled by Log_2(SZ/NC); purple=higher in schizophrenia; green=infinitely high in schizophrenia; blue=higher in healthy controls. Nodes are sized by significance. Significantly changed proteins are indicated by arrows.
