# Supplementary material for: Multi-omics of human plasma reveals molecular features of dysregulated inflammation and accelerated aging in schizophrenia
Source: Mol Psychiatry. 2021 Nov 5;27(2):1217–25. doi: 10.1038/s41380-021-01339-z (PMC9054664; doi:10.1038/s41380-021-01339-z)
Supplement: Supplementary file 2 — Supplemental Table 1 Cohort [file 41380_2021_1339_MOESM2_ESM.pdf]

Supplemental Table 1.
Demographic and Clinical Characteristics of the Sample (*N* = 105)

**Variable**	**HC (*n* = 51)**	**SZ (*n* = 54)**	***t* or *F***	***p*-value**
Sociodemographic Variables				
Age (years)	52.1 (11.5)	53.8 (10.0)	-0.80	0.426
Sex (Female)	49.0%	53.7%	0.23	0.631
Race			1.86	0.762
Caucasian	52.9%	51.9%		
African American	9.8%	14.8%		
Hispanic	31.4%	27.8%		
Asian	5.6%	3.7%		
Other	0.0%	1.9%		
Education			37.26	<0.001
High School or Below	4.1%	61.1%		
Bachelor's Degree	85.7%	35.2%		
Graduate Degree	10.2%	3.7%		
Physical Health				
Smoker (current)	0.0%	55.6%	26.40	<0.001
Substance use at baseline (current)	11.1%	48.1%	15.66	<0.001
Alcohol abuse at baseline (current)	22.0%	44.4%	5.86	<0.05
Antipsychotic medications (currently taking)	0.0%	90.7%	86.77	<0.001
Comorbidity (CIRS-G)	1.9 (2.4)	6.2 (4.4)	-6.05	<0.001
Physical well-being (MOS-36)	52.7 (7.8)	45.4 (9.7)	3.98	<0.001
Mental Health				
Mental well-being (MOS-36)	53.7 (6.1)	43.4 (11.8)	5.35	<0.001
Psychopathology				
Positive symptoms (SAPS)	0.4 (1.1)	5.9 (3.7)	-10.38	<0.001
Negative symptoms (SANS)	1.0 (1.2)	6.2 (4.2)	-8.56	<0.001
Depression (CDRS)	0.8 (1.8)	2.6 (3.3)	-3.49	<0.01
Anxiety (BSIA)	1.4 (2.3)	6.8 (6.1)	-5.72	<0.001

Data are presented as mean (standard deviation) for continuous variables or percent for categorical variables.
BSIA = Brief Symptom Inventory Anxiety Subscale; CDRS = Calgary Depression Scale; CIRS-G = Cummulative Illness Rating Scale for Geriatrics; MOS-36 = Medical Outcomes Survey Short Form; SANS = Scale for the Assessment of Negative Symptoms; SAPS = Scale for the Assessment of Positive Symptoms
